# Knowledge and Practice about Glasgow Coma Scale Assessment among Nurses Working in Adult Intensive Care Units of Federal Public Hospitals in Addis Ababa, Ethiopia: A Cross-Sectional Study

**DOI:** 10.4314/ejhs.v32i5.4

**Published:** 2022-09

**Authors:** Habtamu Andualem, Temesgen Beyene, Wagari Tuli

**Affiliations:** 1 Department of Emergency, St. Peter Hospital, Addis Ababa, Ethiopia; 2 Department of Emergency Medicine, College of Health Sciences, Addis Ababa University, Addis Ababa, Ethiopia; 3 Department of Emergency Medicine, College of Health Sciences, Addis Ababa University, Addis Ababa, Ethiopia

**Keywords:** Glasgow coma scale, knowledge, practice, Intensive Care Unit

## Abstract

**Background:**

The Glasgow Coma Scale is a dependable and objective neurological assessment instrument used for determining and recording a patient's level of consciousness. Therefore, the knowledge, practice, and factors affecting Glasgow coma scale evaluation among nurses working in adult intensive care units of federally administered hospitals in Addis Ababa, Ethiopia, were investigated.

**Methods:**

From April 4 to 24, 2020, 121 Adult Intensive Care Unit nurses at Ethiopian federal hospitals participated in an institutional-based cross-sectional survey with a standardized self-administered questionnaire. The information was entered into Epidata version 3.1 and then exported to SPSS version 25.0 for analysis. Bivariable and multivariable logistic regressions were used to examine the relationships between independent and dependent variables.

**Result:**

According to this study, nurses working in the Adult Intensive Care Unit of federal hospitals in Addis Ababa, Ethiopia, had poor knowledge (51.2%) and poor practice (62%) of the Glasgow Coma Scale's basic theoretical notions and competencies. Furthermore, the education and gender of nurses were linked to their level of knowledge and clinical practice. Being a male and having a master's degree were both significantly linked with knowledge(AOR = 4.13, 95% CI: (1.87–9.1)), (AOR=7.4, 95% CI: (1.4–38)) and practice (AOR = 2.7, 95% CI: (1.2–6)), (AOR = 10.4, 95% CI: (2.0–53)) respectively.

**Conclusion:**

The findings from this study showed that nurses had poor knowledge and application of practice-related clinical scenarios on the Glasgow Coma Scale.

## Introduction

The Glasgow Coma Scale (GCS) is a neurological scale that strives to provide a reliable, objective method of recording a person's conscious state for both initial and follow-up patient assessment. It's the first standardized neurological instrument for determining a patient's level of awareness (1–3).

Teasdale and Jennet first proposed it in 1974 with the goal of standardizing the assessment of the degree of awareness in brain injured patients. It has primarily been used to determine prognosis, compare different patient groups, and monitor neurological state. Its use for the assessment of consciousness in various clinical specialties and research projects has grown over time (4–6). Best eye-opening, best verbal response, and best motor response are the three components of GCS. The scale is numerical, with a total score ranging from 3 to 15. The total score is used to categorize brain injuries into three categories: mild (13–15), moderate (9–12), and severe (3–8; 7,8).

In-patient treatment requires consistent measurement and transmission of the GCS at different periods and amongst different observers. However, actual research showed that when nurses conduct the GCS in a mentoring system, which is an important component of assessment and care for patients suffering from trauma, surgery, or neurological sequelae, they are inconsistent and erroneous (9–11). Nurses working in critical care should employ assessments of awareness level as easily as other routine observations of vital signs (12, 13). They should have the necessary knowledge, abilities, and qualifications to conduct a GCS-based neurological examination (14,15).

Nurses caring for seriously ill patients may encounter scenarios involving any sort of neurologic condition that necessitates GCS evaluation, which is considered a primary nursing function (5, 16). Because of delays in the discovery and treatment of problems, failure to measure GCS is a common cause of preventable mortality and morbidity following a head injury (17, 18). Nurses deal with patients on the front lines of health care. As a result, they should be well-versed in the GCS assessment and confident in their abilities (19, 20). However, several studies assessing nurses' and other clinicians' knowledge of the GCS have revealed a lack of understanding and improper application of this vital instrument (11,21).

The GCS is currently a global challenge in terms of nurse knowledge and clinical practice. For instance, a study conducted in the United States on 217 different emergency care professionals to establish the degree of GCS scoring accuracy found that total GCS rating accuracy for all levels of providers was 33.1 %.Residents were the most accurate in their use of the GCS (51.0%), while nurses were the least accurate (29.0%) (2, 22). Another research evaluating nurses' knowledge in Malaysia, the United Arab Emirates, and Ghana found that 55.56 %, 33.3 %, and 49.4 % of participants, respectively, had low understanding (8, 23, 24).

This lack of knowledge and experience in these areas may impair their ability to make clinical judgments while dealing with unconscious patients(25, 26). As a result, the purpose of this research was to investigate and characterize the current knowledge and practice gap.

## Methods and Materials

**Study area and study period**: The study was conducted in Ethiopia's capital city, Addis Ababa, in federally run facilities. Addis Ababa is situated at approximately 2,400 meters above sea level. In 2007, it covered 290 square kilometers and had a population of 4 million people(27). The city currently has 12 public hospitals and more than 40 private hospitals. Five of the 12 public hospitals in Addis Ababa were administered by federal government (28). This study used four of the federal hospitals by excluding Yeka Kotebe General Hospital because the hospital was used as a Covid center during the study period. The four hospitals were Tikur Anbessa Specialized Hospital (TASH), St. Paul Millenium Medical College and Hospital (SPMMCH), St. Peter Hospital, and the All-Africa Leprosy, Tuberculosis, Rehabilitation, and Training Center (ALERT) hospital are among the facilities involved. There were about 126 nurses working in the four federal hospitals' Adult Intensive Care Units (AICU). The research took place from April 4 through April 24, 2020.

**Study design**: In a cross-sectional study, nurses working at AICU in federally run public hospitals in Addis Ababa, Ethiopia, were assessed on their knowledge, practice, and factors affecting GCS assessment.

**Source population**: All nurses working in Addis Ababa's federally run public hospitals' AICUs.

**Study Population**: Our study population consisted of all nurses working in AICUs of selected federally run public hospitals in Addis Ababa who met the inclusion criteria.

**Inclusion and exclusion criteria**: Nurses with six months or more of work experience who were available during the data collection period were included, whereas nurses on study leave, maternity leave, or annual leave during the study period were excluded.

**Data collection tools and procedure**: This study's questionnaire was derived from prior similar studies (5, 23, 24, 29). The tool was prepared in the English language because all nurses in Ethiopia received their training using the English language. There were three sections to the questionnaire. Respondents' sociodemographic features, understanding of GCS assessment, and GCS assessment practice by nurses were examined. Nurses' demographic features include their gender, age, level of education, job experience, and GCS-related courses or training. Nurses' knowledge of the GCS was assessed using 15 multiple-choice questions with only one correct response. The correct answer received one point, while all other erroneous responses received zero points. Respondents who scored equal or above the mean value for the knowledge questions were considered to have strong knowledge about GCS assessment, whereas those who scored less than the mean value were considered to have poor knowledge. Six clinical scenario practice questions about GCS evaluation were included in the practice section. This question was used to examine how fundamental knowledge is applied in real-life clinical scenarios. A correct response received a one-point score, while an incorrect response received a zero-point score, and the total score was divided into two categories: good clinical practice when respondents answered equal or greater than the mean value for practice questions, and poor clinical practice when respondents answered less than the mean value for practice questions. The data collection was carried out during each nurse's shift. On each shift, one supervisor and one data collector with a BSC in nursing and prior data collection experience were on hand for each hospital to provide consent forms, conduct questionnaires, assist participants with any questions, and collect completed questionnaires to submit to the primary researcher.

**Data quality control**: To verify the quality of the data, the questionnaire was pre-tested two weeks before the actual data collection on nurses working in the ICU at Yekatit 12 hospital, on 5% of the estimated sample for clarity, understandability, and completeness. In addition, data collectors and supervisors received at least a one-day training to improve data quality and verify that all data collectors and assistants had the same information about the study equipment and used the same survey procedures. The data was cleansed for inconsistencies and missing values before being analyzed, and any necessary revisions were assessed.

**Data processing and analysis procedure**: The data was checked, cleaned, and entered into Epidata version 3.1 before being exported to SPSS version 25.0 for analysis. Descriptive statistics were employed to provide an overall and consistent presentation and description of the data during the analysis. The crude significant relationship between each independent variable and the dependent variables was determined using binary logistic regression. During the bivariable analysis, variables with a 95% confidence interval and a P-value of less than 0.25 were incorporated into a multivariable logistic regression analysis to assess the relative influence of confounding variables and the interaction of factors. During a multivariable analysis, a P-value of less than or equal to 0.05 was considered significant.

**Ethical consideration**: Addis Ababa University, College of Health Science, Department of Emergency Medicine ethics committee, provided ethical permission for the study's conduct. Before data collection, clearance letters were granted to federally managed hospitals in Addis Ababa after receiving an official letter from the department. Participants in the study were informed of their ability to leave or not be forced to participate if they did not want to. Participants who were interested in participating in the study were given adequate informed consent by the data collectors, and then written approval was collected from the respondents concerning the study and its objective. The participants' information was kept confidential and was not revealed directly. They were told that there would be no damage and that participating in the study would provide them with a direct benefit.

**Operational definitions**

**Good knowledge**: those respondents who scored the mean and above the mean on knowledge questions.

**Poor knowledge**: those respondents who scored below the mean score on the knowledge question.

**Practice**: is application of theoretical concepts in clinical circumstances or the necessary actions to be taken to help patients

**Good practice**: refers to all AICU nurses who scored at or above the mean of the clinical scenario questionnaire towards GCS assessment.

**Poor practice**: refers to all AICU nurses who scored less than the mean of the clinical scenario questionnaire towards GCS assessment.

## Results

**Socio-demographic characteristics of respondents**: A total of 121 nurses from the four Addis Ababa federally administered hospitals participated in the study, with a 96.0% response rate. The study included 65 (53.7%) males and 56 (46.3%) female nurses. The respondents' ages ranged from 23 to 43 years old, with a mean of 30.07 (SD = 4.862) years. One hundred ten (90.9%) of the participants had a BSc degree, and 11 (9.1%) had an MSc degree (Table 1).

**Table 1:** Socio-demographic characteristics of nurses working at AICU of federal hospitals in Addis Ababa, Ethiopia, 2020 (n=121)

Variables	Categories	Frequency(n)	Percentage (%)
Age	20–24 years	5	4.1
	25–29 years	64	52.9
	30–34 years	24	19.8
	35–39 years	17	14.0
	40 years & above	11	9.1
Sex	Male	65	53.7
	Female	56	46.3
Level of education	BSc degree	110	90.9
	MSc degree	11	9.1
AICU work experience	6 month–2 years	58	47.9
	3–5 years	38	31.4
	6–10 years	13	10.7
	>10 years	12	9.9
Training on GCS	Yes	44	36.4
	No	77	63.6
Work overload	Yes	89	73.6
	No	32	26.4

Knowledge of nurses towards Glasgow Coma Scale assessment: The knowledge questions had a mean score of 53.7%. Respondents who scored equal or above the mean value were thought to be knowledgeable about GCS assessment, while those who scored below the mean value were thought to be less knowledgeable. As a result, AICU nurses had an overall understanding of GCS evaluation of 48.8%. Respondents gave a variety of answers to the specific questions based on their knowledge. Eighty-nine (73.6%) of the study participants responded that the total possible score for the eye component is four (Table 2).

**Table 2:** Knowledge score on each item towards GCS assessment among nurses working at AICU of federal hospitals in Addis Ababa, Ethiopia, 2020 (n=121)

Variables	Correct	Incorrect
	
	Frequency(%%)	Frequency(%)
A total possible score for the eye component of the Glasgow Coma Scale is:	89 (73.6)	32 (26.4)
A total possible score for the verbal component of the Glasgow coma scale is:	74(61.2.)	47 (38.8)
The maximum possible score for the Glasgow Coma Scale is:	85 (70.2)	36 (29.8)
Not a component of the Glasgow Coma Score:	67 (55.4)	54 (44.6)
The minimum score for the Glasgow coma scale is:	63 (52.1)	58 (47.9)
The GCS results that indicate a moderate head injury is between ______ and ______.	52 (42.9)	69 (57.0)
The maximum score on the GCS that correlates with severe traumatic brain injury is:	59 (48.8)	62 (51.2)
Vital signs are a component of Glasgow coma scale.	72(59.5)	49 (40.5)
Component of the GCS which is the least indicator of brain injury severity is:	37 (30.6)	84 (69.4)
When testing the best motor response, you record the response from:	25 (20.7)	96 (79.3)
To test motor response in tetraplegia patients (paralyzed in all four limbs), you will do:	39 (32.2)	82 (67.8)
Patients with a Glasgow Coma Scale score of ______ and below are considered comatose.	50 (41.3)	71 (58.7)
In nursing practice, a reduction of the Glasgow Coma Scale score of _______ is considered as deterioration in conscious level and requires informing the medical team.	69 (57.0)	52 (43.0)
The Glasgow Coma Scale cannot assess intubated patient's level of consciousness.	80 (66.1)	41 (33.9)
You are assessing a road traffic accident patient, who has swollen eyes. You instruct him to open his eyes, but he is unable to. The eye response score is:	25 (20.7)	96 (79.3)

**Practice of nurses towards Glasgow Coma Scale assessment**: The cut point for good and poor practice was determined based on the mean score of correct answers, which was 38.8%. Respondents who scored 38.8% or more were considered good in clinical practice, whereas those who got less than 38.8% were considered poor in clinical practice. As a result, only 38% of the participants were able to answer the mean and greater than mean of the questions correctly. As indicated in Table 3, respondents had variable answers for the specific practical case scenarios.

**Table 3:** Level of practice score on each item towards GCS assessment among nurses working at AICU of federal hospitals in Addis Ababa, Ethiopia, 2020 (n=121)

Variables	Correct	Incorrect
	
	Frequency(%)	Frequency(%)
**Question 1**: An 18-year-old male is hit on the head with a baseball bat. He withdraws & opens his eyes in response to deep painful stimuli. He also mumbles incomprehensively. The GCS is____.	32 (26.4)	89 (73.6)
**Question 2:** An adult unconscious patient flexes his elbow and wrist when pressure is put on the nail bed. However, he does not open his eyes at all and makes grunting noises that are not understood. The GCS is_____.	31 (25.6)	90 (74.4)
**Question 3:** A 40-year-old man is involved in a head-on collision while driving to work. In casualty resuscitation room, he opens his eyes to the pain, is mumbling inappropriately, & tries to stop the medical officer putting a cannula in his arm. The GCS is___.	22 (18.2)	99 (81.8)
**Question 4:** A50-year-old woman jumps from the seventh floor in an attempt to commit suicide. In the casualty resuscitation room, there is no eye-opening or speech. She does not respond when her nail bed is pressed. The GCS is ___.	69 (57.0)	52 (43)
**Question 5:** An adult patient in the ICU is seen to obey simple commands and opens his eyes when he hears you speak. He can talk to you in sentences but seems confused and not sure where he is at present. The GCS is_____.	37 (30.6)	84 (69.4)
**Question 6:** A 31-year-old male is seen in the emergency department & undergoes a quick neurological evaluation following head trauma. He is unable to open his eyes, has no motor movement, & makes no verbal sounds. The GCS is_____.	85 (70.2)	36 (29.8)

**Factors associated with knowledge of nurses about GCS assessment**: Different variables were subjected to bivariable logistic regression analysis. Age, sex, educational level, experience, training, and work overload were all investigated to see if they had a significant relationship with nurses' knowledge of GCS evaluation. To control for confounding factors, all variables with a p-value of less than 0.25 were combined in a multivariable logistic regression. Sex (COR = 3.6, CI = 1.87–9.1, P = 0.001) and educational level (COR = 5.4, CI = 1.12–26, P = 0.036) were shown to have a p-value less than 0.25 in binary logistic regression analysis. Both factors obtained a p-value of less than 0.05 in the multivariable logistic regression. Male nurses were 4 times more likely than female nurses to have strong knowledge (AOR = 4.13, CI = 1.87–9.1, P = 0.001). MSc degree holders were 7.4 times more likely than BSc holders to have high knowledge (AOR=7.4, CI=1.4–38, P=0.018) (Table 4).

**Table 4:** Bivariable and multivariable analysis of factors associated with knowledge towards GCS assessment among nurses working at AICU of federal hospitals in Addis Ababa, Ethiopia, 2020 (n=121)

Variables	Categories	Level of knowledge		COR (95% CI)	p-value	AOR (95% CI)	P-value

		Good N (%)	Poor N (%)				
Sex	Male	41(33.9)	24(19.8)	3.6(1.69–7.7) *	0.001	4.13(1.87–9) **	0.001
	Female	18(14.9)	38(31.4)	1			
Age	20–24	2(1.7)	3(2.5)	0.8(0.9–6.45) *	0.84		
	25–29	36(29.8)	28(23.1)	1.54(0.43–5.6) *	0.51		
	30–34	7(5.8)	17(14.0)	0.49(0.11–2.2) *	0.35		
	35–39	9(7.4)	8(6.6)	1.35(0.29–6.2) *	0.699		
	>= 40	5(4.1)	6(5.0)	1			
Education	MSc degree	9(7.4)	2(1.7)	5.4(1.12–26) *	0.036	7.38(1.4–38) **	0.018
	BSc degree	50(41.3)	60(49.6)	1			
Experience	2moths–2years	29(24.0)	29(24.0)	1.4(0.39–4.9) *	0.6		
	3–5 years	17(14.0)	21(17.4)	1.13(0.30–4.2) *	0.85		
	6–9 years	8(6.6)	5((4.1)	2.24(0.45–11) *	0.32		
	>= 10 years	5(4.1)	7(5.8)	1			
Training	Yes	23(19.0)	21(17.4)	1			
	No	36(29.8)	41(33.9)	0.8(0.38–1.68) *	0.56		
Workload	Yes	43(35.5)	46(38.0)	0.93(0.4–2.09) *	0.87		
	No	16(13.2)	16(13.2)	1			

* COR= Crude Odds Ratio significant at p-value < 0.25 and AOR

** Adjusted Odds Ratio significance at p-< 0.05

**Factors associated with practice of nurses about GCS assessment**: Sex (COR = 2.28, CI = 1.0–4.8, P = 0.033) and respondents' educational level (COR = 8.5, CI = 1.7–41, P = 0.008) were found to have p-values of less than 0.25 in bivariable logistic regression and were then transferred to multivariable logistic regression. According to the output of multivariable logistic regression, the two factors have a p-value of less than 0.05. Male nurses were shown to be 2.7 times more likely than female nurses to have good clinical practice questioning skills (AOR = 2.7, CI = 1.2–6, P = 0.018). MSc degree holders, on the other hand, were found to be 10.4 times more likely than BSc degree holders to have good proficiency in the clinical application of GCS evaluation (AOR = 10.4, CI = 2.0–53, P = 0.005) (Table 5).

**Table 5:** Bivariable and multivariable analysis of factors associated with practice towards GCS assessment among nurses working at AICU of federal hospitals in Addis Ababa, Ethiopia, 2020 (n=121)

Variables	Categories	Level of practice		COR (95% CI)	p-value	AOR (95% CI)	P-value

		Good N (%)	Poor N (%)				
Sex	Female	16(13.2)	40(33.1)	1			
	Male	31(25.6)	34(28.1)	2.28(1.0–4.8) *	0.033	2.7(1.2–6.0) **	0.018
Age	20–24	2(1.7)	3(2.5)	1.8(0.19–16.5) *	0.613		
	25–29	27(22.3)	37(30.6)	1.95(0.47–8.0) *	0.357		
	30–34	7(5.8)	17(14.0)	1.1(0.22–5.4) *	0.908		
	35–39	8(6.6)	9(7.4)	2.37(0.46–12) *	0.300		
	>= 40	3(2.5)	8(6.6)	1			
Education	BSc degree	38(31.4)	72(59.5)	1			
	MSc degree	9(7.4)	2(1.7)	8.53(1.7–41) *	0.008	10.44(2.0–53) **	0.005
Experience	2moth–2years	19(15.7)	39(32.2)	0.49(0.14–1.7) *	0.262		
	3–5 years	14(11.6)	24(19.8)	0.58(0.16–2.2) *	0.42		
	6–9 years	8(6.6)	5(4.1)	1.6(0.33–7.85) *	0.56		
	>= 10 years	6(5.5)	6(5.0)				
Training	Yes	18(14.9)	26(21.5)	1.15(0.5–2.4) *	0.72		
	No	29(24.0)	48(39.7)	1			
Workload	Yes	33(27.3)	56(46.3)	0.76(0.33–1.7) *	0.507		
	No	14(11.6)	18(14.9)	1			

* COR= Crude Odds Ratio significant at p-value < 0.25 and AOR

** Adjusted Odds Ratio significance at p-< 0.05

## Discussion

The GCS assessment for critically sick patients is vital in identifying the advancement or deterioration of mental status in ICU patients. Nurses who work in ICUs should be familiar with and have practice with GCS evaluation. As a result, this study was carried out to analyze nurses' knowledge, practice, and factors influencing GCS evaluation in adult intensive care units of federal public hospitals in Addis Ababa, Ethiopia.

Many of the participants in this study were between the ages of 25 and 29 years old. This result was consistent with findings from a study conducted at the University of Baghdad, which revealed that the majority of participating nurses were between the ages of 28 and 32 years old, and that the majority of them (56.0%) were male (1). The majority of the participants in this study (90.9%) held a bachelor's degree in nursing. This finding is in line with a study conducted in Saudi Arabia which found that the majority of nurses (79.2%) had a bachelor's degree in nursing (11).

According to the findings of this study, 51.2% of nurses had a poor understanding of GCS evaluation. This is in accordance with a study undertaken in the United Arab Emirates and Ghana, which found that 49.7% and 50.4% of staff nurses, respectively, had insufficient knowledge of GCS evaluation (8,24). It is significantly higher than the 33.0% of nurses in a Nigerian tertiary hospital who had inadequate knowledge of GCS assessment (25). These differences could be related to Ethiopian and Nigerian economic and cultural differences.

This study's findings differ from those of a study conducted in Malaysia, which found that 55.56% of nurses had inadequate knowledge of the GCS evaluation (23). Similarly, a study conducted at the University of Baghdad found that all nurses were unfamiliar with the Glasgow coma scale (1). This disparity could be due to differences in the assessment techniques used and the characteristics of the respondents.

The findings of this study on the relationship between knowledge and sex demonstrate that the two variables have a statistically significant relationship such that male nurses were more knowledgeable (P = 0.001). Similar findings were observed that male nurses had much more knowledge than female nurses (8). Another study found that female nurses had much more knowledge than male nurses (24, 30). This disparity could be related to disparities in educational interventions for GCS evaluation between boys and females.

In this study, nurses with postgraduate degrees had superior knowledge of basic theoretical concepts than nurses with bachelor's degrees. A study undertaken in Malaysia and Saudi Arabia revealed the same outcome (11, 23). This demonstrated the importance of continuing education and practice in order to enhance nurses' GCS assessment skills.

The study also found that, while having good knowledge of some of GCS's key theoretical concepts, many nurses are unable to apply that information in clinical circumstances. For example, whereas 48.8% of the participants in this study had a good understanding of the GCS's basic ideas, only 38% of the individuals had good practice of using that information in a clinical situation, indicating that the knowledge could not be transferred into practice. This is in line with a study conducted in Vietnam, which found that the majority of nurses (> 90%) replied correctly to questions about their GCS basic knowledge; yet, only 47.9% of nurses correctly answered questions about clinical scenarios requiring the application of basic knowledge(30). In a study conducted in Ghana, 62.6% of the participants had good basic theoretical knowledge about GCS, but only 5.2% had good expertise in the application of this basic theoretical information in practice relevant clinical scenario questions (24). In contrast, research done in India indicated that only 16.36% of people had insufficient skills (31). The disparities in this study could be due to variances in the instruments, settings, and quality of training obtained by the respondents.

The findings of this study on the relationship between clinical practice and sex demonstrate that the two variables have a statistically significant relationship and that male nurses had good practical skills (P = 0.018). Female nurses, on the other hand, demonstrated statistically considerably higher levels of understanding of the GCS with its application of practice related clinical scenario questions in the clinical situation, according to the findings of a study done in Ghana (24). It's possible that the finding has something to do with sample size.

In this study, education was found to be substantially linked with clinical practice toward GCS evaluation. It was found that master's holders had good practice (P = 0.005). It is in line with a study done in Egypt which stated that there is a highly statistically significant improvement in the level of competence of nurses' practice in regards to GCS, which was noted immediately after the educational program was implemented compared to before the educational program. The more GCS knowledge the nurses' personnel had, the more correctly they could practice (14).

In conclusion, even though the majority of the participants had poor knowledge of basic theoretical concepts of the GCS and demonstrated poor practice on the application of the basic knowledge in clinical scenarios, the study might help academicians, clinicians, and policy makers to have better understanding of the GCS application. These findings suggest that nurses were not able to incorporate their theoretical knowledge of the GCS with its practical application in the clinical situation. Therefore, providing in-service training for AICU nurses regarding the use of GCS at each hospital ICU was recommended by researchers.

## References

[1] Jaddoua BA, Mohammed WK, Abbas AD (2013). Assessment of nurse's knowledge concerning Glasgow coma scale in neuro surgical wards. Kufa J Nurs Sci.

[2] Al-Quraan H, AbuRuz ME (2016). Assessment of Jordanian nurses' knowledge to perform Glasgow Coma Scale. European Scientific Journal.

[3] Edwards SL (2001). Using the Glasgow Coma Scale: analysis and limitations. British Journal of Nursing.

[4] Matis G, Birbilis T (2008). The Glasgow Coma Scale-a brief review Past, present, future. Acta Neurol Belg.

[5] Ehwarieme T, Anarado A (2017). Nurses use of Glasgow coma scale in neurological assessment of patients in university of Benin Teaching Hospital, Benin City Edo State, Nigeria. Journal of Medicine and Biomedical Research.

[6] Teasdale GM, Allan D, Brennan P, McElhinney E, Mackinnon L (2014). Forty years on: updating the Glasgow Coma Scale. Nursing Times.

[7] Bhaskar S (2017). Glasgow coma scale: Technique and Interpretation. Clinics in Surgery.

[8] Ahmed Y, Ahmed S, Mohammad A, Eida S, Hamid Y, Nezar A (2018). Assessment of Nurses Knowledge About Glasgow Coma Scale at al Dhafra Hospitals, Abu Dhabi, United Arab Emirates. J Clin Rev Case Rep.

[9] Reith FC, Brennan PM, Maas AI, Teasdale GM (2016). Lack of standardization in the use of the Glasgow Coma Scale: results of international surveys. Journal of neurotrauma.

[10] Iankova A (2006). The Glasgow Coma Scale clinical application in emergency departments. Emergency Nurse.

[11] Albougami A (2019). Exploring nurses ‘knowledge of the Glasgow Coma Scale in intensive care and emergency departments at a tertiary hospital in Riyadh city, Saudi Arabia. The Malaysian Journal of Nursing (MJN).

[12] Middleton PM (2012). Practical use of the Glasgow Coma Scale; a comprehensive narrative review of GCS methodology. Australasian Emergency Nursing Journal.

[13] Palmer R, Knight J (2006). Assessment of altered conscious level in clinical practice. British journal of nursing.

[14] Ahamed ST, Ebraheim MN (2017). Glasgow Coma Scale Technique: Effect of Theoretical and Practical Educational Program on Nurses' Compliance. Journal of Nursing and Health Science.

[15] Oktarina Y, Simajuntak CA (2018). Comparison of Glasgow Coma Scale with full outline of unresponsiveness score in measuring consciousness level of endotracheal tube intubated patient in the intensive care unit. International Journal of Research in Medical Sciences.

[16] Santos WC, Vancini-Campanharo CR, Lopes MCBT, Okuno MFP, Batista REA (2016). Assessment of nurse's knowledge about Glasgow coma scale at a university hospital. Einstein (São Paulo).

[17] Chan MF, Mattar I, Taylor BJ (2013). Investigating factors that have an impact on nurses' performance of patients' conscious level assessment: a systematic review. Journal of Nursing Management.

[18] Chan MF, Matter I (2013). Investigating nurses' knowledge, attitudes and self-confidence patterns to perform the conscious level assessment: A cluster analysis. International Journal of Nursing Practice.

[19] Jennett B (2005). Development of Glasgow coma and outcome scales. Nepal Journal of Neuroscience.

[20] AbuRuz ME (2016). Simplifying Glasgow Coma Scale Use for Nurses. International journal of Nursing Didactics.

[21] Summers C, McLeod A (2017). What influences nurses to undertake accurate assessment of the Glasgow Coma Scale?. British Journal of Neuroscience Nursing.

[22] Bledsoe BE, Casey MJ, Feldman J, Johnson L, Diel S, Forred W (2015). Glasgow Coma Scale scoring is often inaccurate. Prehospital and disaster medicine.

[23] Singh B, Chong MC, Zakaria MIb, Cheng ST, Tang LY, Azahar NH (2016). Assessing nurses' knowledge of Glasgow coma scale in emergency and outpatient department. Nursing research and practice.

[24] Alhassan A, Fuseini A-G, Musah A (2019). Knowledge of the Glasgow Coma Scale among nurses in a tertiary hospital in Ghana. Nursing research and practice.

[25] Ehwarieme TA, Anarado A (2016). Nurses' knowledge of Glasgow Coma Scale in neurological assessment of patients in a selected tertiary hospital in Edo State, Nigeria. Africa journal of nursing and midwifery.

[26] Emejulu J, Nkwerem S, Ekweogwu O (2014). Assessment of physicians' knowledge of Glasgow coma score. Nigerian journal of clinical practice.

[27] Data B (2007). Ethiopia and its capital, Addis Ababa. Ethiopia's General Profile.

[28] Addis Ababa's ailing state hospitals - Addis Standard [Internet].

[29] Bansal S, Chawla R (2016). Awareness of Glasgow Coma Scale in anesthesiology post-graduates in India: A survey. Journal of Neuro anesthesiology and Critical Care.

[30] Nguyen TH (2011). The accuracy of Glasgow coma scale knowledge and performance among Vietnamese nurses.

[31] Teles M, Bhupali P, Madhale M (2013). Effectiveness of self-instructional module on knowledge and skills regarding use of Glasgow coma scale in neurological assessment of patients among nurses working in critical care units of KLE Dr. Prabhakar Kore hospital and medical research center, Belgaum. Journal of Krishna Institute of Medical Sciences University.

